# The combined use of vitamin B1 and vitamin B12 accelerates the recovery of gastrointestinal function after rectal cancer surgery

**DOI:** 10.3389/fnut.2025.1658150

**Published:** 2025-10-23

**Authors:** Song Tang, Simin Luo, Weikun Fang, Zhenyu Chen, Yongfang Liao, Huiqiang Cai, Weibin Zhang, Dan Chen

**Affiliations:** ^1^Department of Gastrointestinal Surgery, The First Affiliated Hospital of Guangdong Pharmaceutical University, Guangzhou, Guangdong, China; ^2^Breast Tumor Center, Sun Yat-sen Memorial Hospital, Sun Yat-sen University, Guangzhou, Guangdong, China; ^3^Department of Surgery, The First Affiliated Hospital of Guangdong Pharmaceutical University, Guangzhou, Guangdong, China; ^4^Department of General Surgery, Dianbai District People’s Hospital, Maoming, Guangdong, China

**Keywords:** vitamin B1, vitamin B12, gastrointestinal function, postoperative recovery, rectal cancer

## Abstract

**Background:**

The recovery of gastrointestinal function after rectal cancer surgery is a key factor influencing patients’ postoperative quality of life. This study is the first to explore the role of vitamin B1 and B12 in the recovery of gastrointestinal function after rectal cancer surgery.

**Methods:**

Eligible patients were divided into four groups: no vitamin group, vitamin B1 alone group, vitamin B12 alone group, and combined vitamin B1 and B12 group. Univariate analysis was used to compare the differences in the time to first flatus among the four groups. Due to the small number of patients using only vitamin B1 or only vitamin B12, these patients were excluded, and univariate and multivariate linear regression analyses were performed on the time to first flatus.

**Results:**

The time to first flatus exhibited a non-normal distribution. The Kruskal-Wallis rank sum test indicated significant differences in the time to first flatus among the four groups (*p* = 0.0152). However, Dunn’s pairwise comparison test showed that only the combined vitamin B1 and B12 group differed significantly from the no vitamin group (*p* = 0.001). Univariate linear regression analysis demonstrated that intraoperative blood loss (*p* = 0.001), enterostomy (*p* = 0.002), ileostomy (*p* < 0.001), and combined use of vitamin B1 and B12 (*p* = 0.007) significantly affected the time to first flatus. After removing variable with severe multicollinearity, the results of the multivariate regression analysis showed that intraoperative blood loss (*p* = 0.001), ileostomy (*p* = 0.001), and combined use of vitamin B1 and B12 (*p* = 0.026) still had significant effects on the time to first flatus.

**Conclusion:**

The combined use of vitamin B1 and B12 can accelerate the recovery of gastrointestinal function after rectal cancer surgery.

## Introduction

Colorectal cancer is the third most common cancer and the third leading cause of cancer-related mortality, with rectal cancer accounting for approximately one-third of cases (1). Despite significant advances in neoadjuvant therapy in recent years, surgery remains the only curative treatment for rectal cancer (2). The postoperative recovery of gastrointestinal function is a key factor influencing the quality of postoperative rehabilitation. If gastrointestinal function recovers quickly, patients can resume oral intake early, replenish nutrients, and enhance their confidence and physical strength. However, delayed gastrointestinal function recovery may lead to nausea, vomiting, abdominal distension, or even bowel obstruction, exacerbating patient suffering, increasing the burden, and potentially prolonging hospital stays.

Previous studies have shown that the use of dexmedetomidine during anesthesia, chewing gum postoperatively, administration of probiotics during the perioperative period, and non-steroidal anti-inflammatory drugs can accelerate the recovery of gastrointestinal function after abdominal surgery (3–9). However, the existing methods and their effects remain limited, with various constraints that cannot fully meet the clinical needs of gastrointestinal surgery. Previous studies have shown that nutrition and metabolism play important roles in colorectal cancer (10, 11). Vitamin B1 and B12, as part of the B-vitamin family, have not been explicitly reported in the literature regarding their role in postoperative gastrointestinal function recovery. Nevertheless, based on long-term empirical observations in our department, vitamin B1 and B12 may have potential benefits in promoting gastrointestinal function recovery.

To further explore the role of vitamin B1 and B12 in gastrointestinal function recovery, we selected rectal cancer as a complex disease model. Rectal cancer surgery involves key surgical factors such as enterostomy and sphincter preservation, which can reflect the practical application value of vitamin B1 and B12 in gastrointestinal function recovery. Through univariate and multivariate analyses of patient data, we comprehensively verified for the first time the effect of vitamin B1 and B12 on postoperative gastrointestinal function recovery. With this study, we aim to provide new therapeutic insights for optimizing postoperative gastrointestinal function recovery, particularly in utilizing simple and low-cost treatments such as vitamin B1 and B12, thereby promoting their clinical application.

## Methods

### Patient data selection

This study is a retrospective cohort study, selecting data from rectal cancer patients treated at the Second Department of General Surgery (Gastrointestinal Surgery) in our hospital between 2015 and 2024. Inclusion criteria were: (1) rectal cancer patients who underwent surgical treatment; (2) complete medical records with detailed documentation of the first postoperative flatus time. Exclusion criteria were: (1) rectal cancer patients receiving conservative treatment; (2) incomplete medical records with unavailable accurate postoperative first flatus time.

### Outcome measure

The first postoperative flatus time is an intuitive and easily observed indicator that effectively reflects gastrointestinal function recovery, previously used as a primary outcome measure in several studies (6). Although postoperative defecation time is also commonly used as an indicator of gastrointestinal function recovery, it may not accurately represent gastrointestinal recovery in rectal cancer patients, especially those with bowel obstruction or stomas. The incidence of postoperative bowel obstruction with clear radiological evidence is low in our department, making it an unreliable statistical indicator. Bowel sounds have been previously reported as an unreliable outcome measure for bowel recovery (12). Furthermore, postoperative hospital stay is influenced by various factors, including non-gastrointestinal complications. Therefore, to more precisely assess the effects of vitamin B1 and B12 on gastrointestinal recovery, this study uses the first postoperative flatus time as the sole outcome measure. The first flatus time in this study includes flatus through the anus, ileostomy, or colostomy. For convenience, flatus time is rounded to the nearest whole number in days.

### Use of vitamin B1 and vitamin B12

None of the patients received vitamin B1 or vitamin B12 supplementation before surgery. Postoperative patients began using vitamin B1 and vitamin B12 upon returning to the ward. The specific regimen was: (1) Vitamin B1 injection, 0.1 g, intramuscular injection, once daily; (2) Vitamin B12 injection, 500 μg, intramuscular injection, once daily. Other treatments followed standardized postoperative protocols. According to the use of vitamin B1 and B12, patients were divided into four groups: no vitamin group, vitamin B1 alone group, vitamin B12 alone group, and combined vitamin B1 and B12 group.

### Patient information

To comprehensively investigate the effects of vitamin B1 and B12 on postoperative gastrointestinal recovery in rectal cancer patients, baseline and perioperative data were collected. Baseline data included gender, age, body mass index (BMI), American Society of Anesthesiologists (ASA) classification, smoking status, alcohol consumption, and comorbidities such as hypertension, coronary heart disease (CHD), or diabetes. Perioperative data included preoperative, intraoperative, and postoperative information. Preoperative data comprised the distance of the tumor from the anal verge, hemoglobin level, albumin level, presence of bowel obstruction, neoadjuvant therapy (chemotherapy, targeted therapy, immunotherapy, and radiotherapy), and neoadjuvant radiotherapy status. Intraoperative data included the type of surgery, combination with other gastrointestinal surgeries, operation duration, blood loss, presence of stoma, and stoma type. Postoperative data included TNM staging, first postoperative flatus time, and the use of vitamin B1 and B12.

### Data analysis

All data analyses were performed using R Studio software (R version 4.3.1). First, univariate analysis was conducted to compare the differences in first postoperative flatus time among the four groups. Subsequently, univariate and multivariate linear regression analyses were used to evaluate the effect of combined use of vitamin B1 and B12 on first postoperative flatus time. During the analysis, we also compared the clinic opathological characteristics between the combined vitamin group and the no vitamin group, and performed multicollinearity checks for the variables included in the multivariate linear regression.

## Results

### Comparison among the four groups

A total of 167 patients were included in the study. Among them, 123 patients were in the no-vitamin group, 13 patients in the vitamin B1-only group, 7 patients in the vitamin B12-only group, and 24 patients in the combined vitamin B1 and B12 group. Only 5 patients had postoperative bowel obstruction confirmed by imaging evidence, including 4 patients in the no-vitamin group and 1 patient in the vitamin B12-only group.

**Figure 1:**
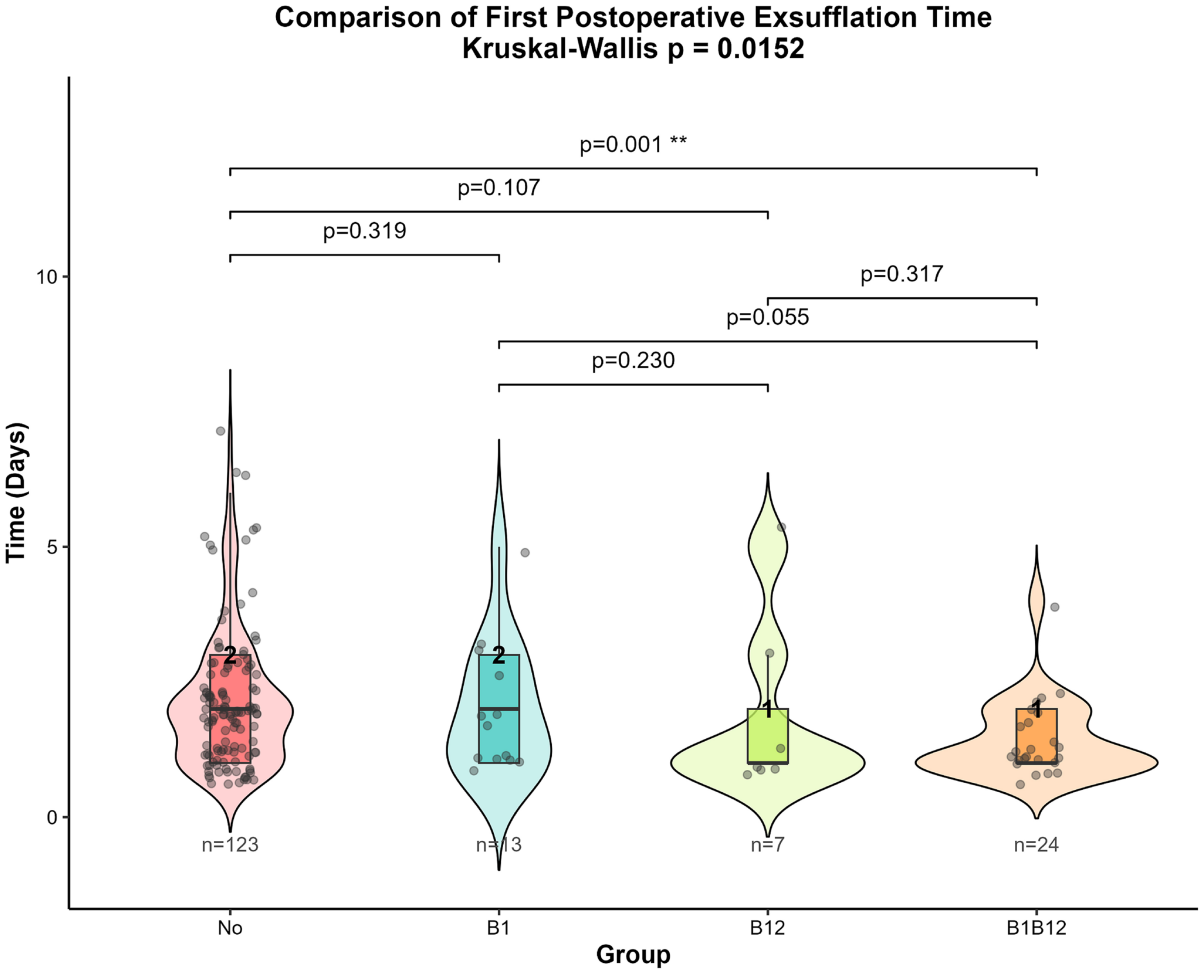
Kruskal-Wallis rank sum test and Dunn’s test results for time to first postoperative flatus among the four groups.

As the time to first postoperative flatus was non-normally distributed, we first performed the Kruskal-Wallis rank sum test and Dunn’s test to compare the differences in time to first postoperative flatus among the four groups. As shown in Figure 1, the Kruskal-Wallis rank sum test yielded a *p*-value of 0.0152, indicating a statistically significant difference among the four groups. Subsequently, Dunn’s pairwise comparison test (Figure 1) revealed a significant difference between the combined vitamin B1 and B12 group and the no-vitamin group (*p* = 0.001).

### Univariate linear regression

After excluding patients from the vitamin B1 alone group and the vitamin B12 alone group, 147 patients remained. The distribution of clinicopathological characteristics between the two groups is shown in Supplementary Table 1, with significant differences observed only in ASA classification, intraoperative blood loss and hypertension (*p* < 0.05). Postoperative time to first flatus was used as the dependent variable, while baseline and perioperative data were used as independent variables for univariate linear regression analysis. The results showed that intraoperative blood loss, presence of stoma, stoma type (ileostomy), and combined use of vitamin B1 and vitamin B12 significantly affected postoperative time to first flatus. No other variables showed statistical significance. The results are shown in Table 1.

**Table 1:** Results of univariate and multivariate linear regression analysis of the first postoperative flatus time.

Variable	Univariate linear regression *β*		Multiple linear regression *β*	
	(95%CI)	*p* value	(95%CI)	*p* value
Baseline data				
Sex (male vs. female)	−0.129 (−0.537, 0.278)	0.534		
Age (year)	0.003 (−0.014, 0.020)	0.728		
BMI (kg/m^2^)	0.030 (−0.029, 0.089)	0.316		
ASA (II vs. I)	0.223 (−0.337, 0.782)	0.441		
ASA (III vs. I)	−0.413 (−1.004, 0.178)	0.173		
Smoke (yes vs. no)	0.116 (−0.319, 0.551)	0.598		
Alcohol (yes vs. no)	0.305 (−0.233, 0.843)	0.271		
Hypertension (yes vs. no)	0.150 (−0.248, 0.548)	0.461		
CHD (yes vs. no)	−0.395 (−0.983, 0.193)	0.189		
Diabetes (yes vs. no)	0.248 (−0.244, 0.740)	0.324		
Perioperative data				
Tumor distance (cm)	0.042 (−0.006, 0.090)	0.094		
Hemoglobin (g/L)	−0.002 (−0.012, 0.007)	0.660		
Albumin (g/L)	−0.009 (−0.056, 0.038)	0.712		
Ileus (yes vs. no)	−0.764 (−1.669, 0.140)	0.098		
Neoadjuvant therapy (yes vs. no)	−0.094 (−0.553, 0.365)	0.688		
Neoadjuvant radiotherapy (yes vs. no)	0.037 (−0.503, 0.577)	0.893		
Operation type (miles vs. dixon)	−0.015 (−0.683, 0.654)	0.964		
Combined surgery (yes vs. no)	−0.188 (−1.174, 0.797)	0.707		
Time (min)	0.001 (−0.002, 0.003)	0.185		
Blood loss (mL)	0.002 (0.001, 0.004)	**0.001**	0.002 (0.001, 0.003)	**0.001**
Enterostomy (yes vs. no)	−0.652 (−1.069, −0.235)	**0.002**		
Enterostomy type (ileum vs. no)	−0.879 (−1.360, −0.397)	**<0.001**	−0.776 (−1.238, −0.314)	**0.001**
Enterostomy_type (colon vs. no)	−0.200 (−0.843, 0.443)	0.542	−0.264 (−0.876, 0.349)	0.401
TNM (II vs. I)	0.200 (−0.351, 0.751)	0.478		
TNM (III vs. I)	0.357 (−0.184, 0.900)	0.198		
TNM (IV vs. I)	0.200 (−0.480, 0.880)	0.564		
Combining vitamins (yes vs. no)	−0.713 (−1.224, −0.202)	**0.007**	−0.559 (−1.046, −0.072)	**0.026**

*β* is the regression coefficient. BMI, body mass index; ASA, American society of anesthesiologists; CHD, coronary heart disease, Tumor distance, Distance between tumor and anal verge. Miles is an abdominoperineal radical resection of rectal cancer, and Dixon is a transabdominal resection of rectal cancer. Bold p-values indicate statistical significance (*p* < 0.05).

### Multivariate linear regression

**Figure 2:**
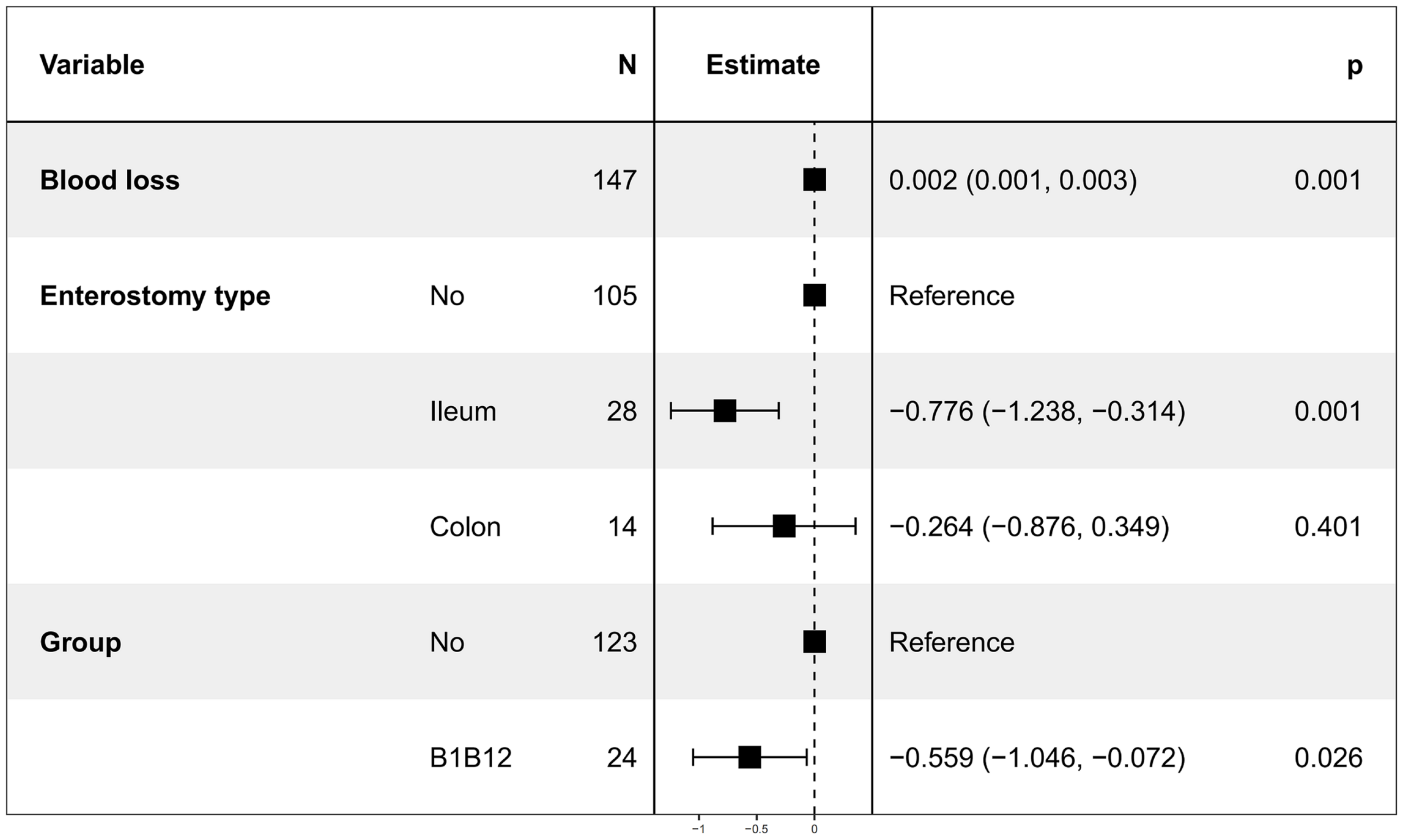
Results of multivariate linear regression for first postoperative flatus time.

We continued to use time to first postoperative flatus as the dependent variable. Due to severe multicollinearity between enterostomy and enterostomy type, only enterostomy type was retained. Multivariate linear regression was then performed using intraoperative blood loss [Variance Inflation Factor (VIF) = 1.0017], enterostomy type (VIF = 1.0077), and combined use of vitamin B1 and B12 (VIF = 1.0157) as independent variables. The results (Figure 2) showed that intraoperative blood loss, ileostomy, and combined use of vitamin B1 and B12 remained significantly associated with time to first postoperative flatus (*p* < 0.05), while colostomy had no statistically significant effect (Table 1).

## Discussion

Rectal cancer accounts for about one-third of colorectal cancers, making it the eighth most common cancer worldwide and the tenth leading cause of cancer-related deaths (1, 13). Surgery remains the only curative treatment for rectal cancer. Total mesorectal excision has become the gold standard for rectal cancer surgery (14). For patients with low rectal cancer, two surgical approaches are available: one is abdominal rectal cancer resection with sphincter preservation, also known as anterior resection, and the other is abdominoperineal resection, which often requires the establishment of a permanent colostomy (15). However, regardless of the chosen surgical method, the recovery of gastrointestinal function is critical to the overall postoperative recovery of patients, especially in the era advocating for enhanced recovery after surgery.

Postoperative gum chewing has been widely proven to accelerate the recovery of gastrointestinal function in abdominal and orthopedic surgeries (3–5, 16, 17). However, the specific mechanism is not yet fully clear. Previous studies suggest that chewing gum stimulates the head-vagus nerve, promotes the secretion of gastrointestinal hormones, and thus stimulates gastrointestinal motility, as well as promotes the secretion of pancreatic juice and saliva, creating a “sham feeding” effect (18). Furthermore, certain types of gum, particularly sugar-free gum, contain ingredients like sorbitol, which may have a laxative effect (18). However, in previous studies, certain exclusion criteria have been set for postoperative gum chewing, such as excluding individuals with a history of abdominal surgery, children, patients with intraoperative or postoperative complications, and common comorbidities such as diabetes (17). This has limited the widespread applicability of this method.

Dexmedetomidine, an intraoperative anesthetic sedative, has been shown to accelerate the recovery of gastrointestinal function (8, 9). However, meta-analyses point out that the evidence is weak and there is a lack of clear dosage recommendations (9). Nonsteroidal anti-inflammatory drugs (NSAIDs) have also been shown to promote gastrointestinal recovery, but existing evidence is insufficient to determine whether selective or non-selective drugs are preferable (7). Additionally, NSAIDs may increase gastric mucosal injury (19). For complex surgeries like rectal cancer, this undoubtedly increases the risk of stress-related gastric ulcers. Some studies have shown that *Bacillus subtilis* TBC169 probiotics can accelerate postoperative gastrointestinal recovery in gynecological laparoscopic surgery patients (6). However, there are multiple limitations in these studies, such as excluding patients with a history of intestinal surgery and those with long operative times.

Given the limitations of current methods and their limited effectiveness, existing treatments do not fully meet the needs of gastrointestinal surgery clinical practice. Therefore, more simple, cost-effective, and widely applicable options need to be explored. In long-term clinical practice, we observed that vitamins B1 and B12 may have potential benefits in promoting gastrointestinal recovery. Based on this, we conducted a retrospective cohort analysis focusing on the complex condition of rectal cancer surgery.

We first performed univariate analysis on the four groups. The results showed that the *p*-value from the Kruskal-Wallis test was 0.0152, suggesting significant differences in the first postoperative flatus time among the four groups. However, paired comparisons from the Dunn test indicated that only the group receiving both vitamin B1 and B12 showed a significant difference compared to the no-vitamin group (*p* = 0.001). This may indicate that vitamin B1 and B12 have a synergistic effect, which is not significant when used alone.

After excluding patients who received only vitamin B1 or only vitamin B12, and considering that gastrointestinal recovery may be influenced by patients’ physical conditions and perioperative procedures, we performed univariate and multivariate linear regression analysis on the first postoperative flatus time. Linear regression was chosen because all patients experienced the event before discharge, the outcome was measured in days, and the data had moderate dispersion, allowing for straightforward interpretation of covariate effects on mean flatus time. Univariate linear regression results showed that intraoperative blood loss, enterostomy, ileostomy, and combined use of vitamins B1 and B12 significantly influenced the time to first postoperative flatus. After removing variables with severe multicollinearity, the results of the multivariate linear regression analysis showed that intraoperative blood loss, ileostomy, and combined use of vitamin B1 and B12 still had significant effects on the time to first flatus. Specifically, increased intraoperative blood loss prolonged the time to first flatus, whereas ileostomy and the combined use of vitamin B1 and B12 significantly shortened it.

The impact of intraoperative blood loss on first flatus time has been reported in previous studies. Extensive blood loss can exacerbate the sympathetic and endocrine stress responses, leading to more intestinal injury and inflammation, thus delaying gastrointestinal recovery (20). Although ileostomy and colostomy did not show significant differences in multivariate regression analysis, they still have some plausibility from a practical standpoint. Generally, small bowel peristalsis recovers the fastest after surgery (within 24 h), followed by the stomach (24–48 h), and then the large intestine (48–72 h) (21, 22). Therefore, a stoma, especially an ileostomy, can shorten the distance from the small intestine to the stoma, which may explain why a significant difference was observed for ileostomy in the analysis.

The mechanism by which combined use of vitamins B1 and B12 promotes gastrointestinal recovery is currently unclear. Both vitamins are B vitamins. Studies have shown that B vitamins are associated with gut microbiota (23). Vitamin B12, also known as cobalamin, is synthesized and utilized by bacteria in the human gut microbiome and may be related to changes in gut bacterial abundance and diversity (24–26). Vitamin B12 injection is commonly used to treat B12 deficiency in patients with malabsorption (24). Additionally, supplementing vitamin B12 may benefit gastrointestinal diseases through its interaction with the gut microbiota (24). Vitamin B1, also known as thiamine, cannot be synthesized endogenously in humans, and its supply is entirely dependent on dietary intake (27). Thiamine deficiency can occur in gastrointestinal surgery, malnutrition, refeeding syndrome, and alcoholism (28). Thiamine deficiency inhibits pancreatic acinar cells, significantly reducing digestive enzyme secretion (29). Some studies also suggest that thiamine can alleviate symptoms of abdominal pain, nausea, vomiting, and anorexia (30, 31). In terms of safety, both vitamins B1 and B12 are water-soluble vitamins with short half-lives and are rapidly excreted from the body (32, 33).

Although our study is the first to demonstrate that combined use vitamin B1 and B12 can accelerate postoperative gastrointestinal recovery in patients with rectal cancer, several limitations should be acknowledged. First, the number of patients receiving vitamin B1 or B12 alone was small, and these groups were excluded from the primary analysis, which may introduce potential bias. Therefore, larger studies are needed to clarify the independent effects of vitamin B1 and B12. Second, the precise mechanisms by which the combination of vitamin B1 and B12 promotes gastrointestinal recovery remain unclear and warrant further basic and clinical investigation. Third, as this was a retrospective study, the recording of time to first postoperative flatus was not standardized; for convenience, the values were rounded, which may reduce precision and introduce some error. Finally, although no patients received vitamin B1 or B12 preoperatively, their baseline levels were not routinely measured in prior clinical care, highlighting the need for future prospective studies to assess this factor more comprehensively.

## Conclusion

The combined use of vitamins B1 and B12 offers a new, simple, cost-effective, and safe treatment option for promoting gastrointestinal recovery after rectal cancer surgery, but further research is needed to establish its broad clinical applicability and elucidate its mechanism of action.

## Data Availability

The original contributions presented in the study are included in the article/Supplementary material, further inquiries can be directed to the corresponding authors.

## References

[1] Siegel RL Miller KD Fuchs HE Jemal A. Cancer statistics, 2022. CA Cancer J Clin. (2022) 72:7–33. doi: 10.3322/caac.21708, PMID: 35020204

[2] Keller DS Berho M Perez RO Wexner SD Chand M. The multidisciplinary management of rectal cancer. Nat Rev Gastroenterol Hepatol. (2020) 17:414–29. doi: 10.1038/s41575-020-0275-y, PMID: 32203400

[3] Liu Q Jiang H Xu D Jin J. Effect of gum chewing on ameliorating ileus following colorectal surgery: a meta-analysis of 18 randomized controlled trials. Int J Surg (London, England). (2017) 47:107–15. doi: 10.1016/j.ijsu.2017.07.107, PMID: 28867465

[4] Mei B Wang W Cui F Wen Z Shen M. Chewing gum for intestinal function recovery after colorectal cancer surgery: a systematic review and meta-analysis. Gastro Res Pract. (2017) 2017:3087904. doi: 10.1155/2017/3087904 PMC5651113 29312450

[5] Pereira Gomes Morais E Riera R Porfírio GJ Macedo CR Sarmento Vasconcelos V de Souza Pedrosa A. Chewing gum for enhancing early recovery of bowel function after caesarean section. Cochrane Database Syst Rev. (2016) 10:Cd011562. doi: 10.1002/14651858.CD011562.pub2 27747876 PMC6472604

[6] Li Z Guan Z Bai N Yan Y Niu Z Xu J. *Bacillus coagulans* TBC169 probiotics for the recovery of intestinal function after gynecological laparoscopic surgery: a randomized, placebo-controlled trial. Int J Clin Pharm. (2022) 44:1287–95. doi: 10.1007/s11096-022-01451-2, PMID: 35882823

[7] Milne TGE Jaung R O'Grady G Bissett IP. Nonsteroidal anti-inflammatory drugs reduce the time to recovery of gut function after elective colorectal surgery: a systematic review and meta-analysis. Colorectal Dis. (2018) 20:O190–8. doi: 10.1111/codi.14268, PMID: 29781564

[8] Wu Y Cai Z Liu L Wang J Li Y Kang Y. Impact of intravenous dexmedetomidine on gastrointestinal function recovery after laparoscopic hysteromyomectomy: a randomized clinical trial. Sci Rep. (2022) 12:14640. doi: 10.1038/s41598-022-18729-0, PMID: 36030343 PMC9420113

[9] Behera BK Misra S Jena SS Mohanty CR. The effect of perioperative dexmedetomidine on postoperative bowel function recovery in adult patients receiving general anesthesia. Minerva Anestesiol. (2022) 88:51–61. doi: 10.23736/S0375-9393.21.15773-6 34527407

[10] Crudele L De Matteis C Novielli F Petruzzelli S Di Buduo E Graziano G. Fasting hyperglycaemia and fatty liver drive colorectal cancer: a retrospective analysis in 1145 patients. Intern Emerg Med. (2024) 19:1267–77. doi: 10.1007/s11739-024-03596-6, PMID: 38668822 PMC11364717

[11] De Matteis C Crudele L Gadaleta RM Di Buduo E Novielli E Petruzzelli S. Low adherence to Mediterranean diet characterizes metabolic patients with gastrointestinal cancer. Nutrients. (2024) 16:630. doi: 10.3390/nu16050630, PMID: 38474758 PMC10933917

[12] Read TE Brozovich M Andujar JE Ricciardi R Caushaj PF. Bowel sounds are not associated with flatus, bowel movement, or tolerance of oral intake in patients after major abdominal surgery. Dis Colon Rectum. (2017) 60:608–13. doi: 10.1097/DCR.0000000000000829, PMID: 28481855

[13] Sung H Ferlay J Siegel RL Laversanne M Soerjomataram L Jemal A. Global Cancer statistics 2020: GLOBOCAN estimates of incidence and mortality worldwide for 36 cancers in 185 countries. CA Cancer J Clin. (2021) 71:209–49. doi: 10.3322/caac.21660 33538338

[14] Enker WE. Total mesorectal excision--the new golden standard of surgery for rectal cancer. Ann Med. (1997) 29:127–33. doi: 10.3109/07853899709113698, PMID: 9187227

[15] Herrinton LJ Altschuler A McMullen CK Bulkley JE Hornbrook MC Sun V. Conversations for providers caring for patients with rectal cancer: comparison of long-term patient-centered outcomes for patients with low rectal cancer facing ostomy or sphincter-sparing surgery. CA Cancer J Clin. (2016) 66:387–97. doi: 10.3322/caac.21345, PMID: 26999757 PMC5618707

[16] Liao XQ Li SL Peng YC Chen LW Lin YJ. Effects of chewing gum on gastrointestinal function in patients following spinal surgery: a meta-analysis and systematic review. Eur Spine J. (2022) 31:2536–46. doi: 10.1007/s00586-022-07304-w, PMID: 35852608

[17] Short V Herbert G Perry R Atkinson C Ness AR Penfold C. Chewing gum for postoperative recovery of gastrointestinal function. Cochrane Database Syst Rev. (2015) 2015:Cd006506. doi: 10.1002/14651858.CD006506.pub3, PMID: 25914904 PMC9913126

[18] Tandeter H. Hypothesis: hexitols in chewing gum may play a role in reducing postoperative ileus. Med Hypotheses. (2009) 72:39–40. doi: 10.1016/j.mehy.2008.06.044, PMID: 18783895

[19] Igarashi M Takagi A Jiang X Hasumi K Watanabe S Deguchi R. Analysis of *helicobacter pylori* and nonsteroidal anti-inflammatory drug-induced gastric epithelial injury. Aliment Pharmacol Ther. (2002) 16:235–9. doi: 10.1046/j.1365-2036.16.s2.6.x 11966547

[20] Artinyan A Nunoo-Mensah JW Balasubramaniam S Gauderman J Essani R Gonzalez-Ruiz C. Prolonged postoperative ileus-definition, risk factors, and predictors after surgery. World J Surg. (2008) 32:1495–500. doi: 10.1007/s00268-008-9491-2, PMID: 18305994

[21] Nimarta Singh NV Shruti Gupta RJ. Effectiveness of chewing gum on bowel motility among the patients who have undergone abdominal surgery. Int J Midwifery Nurs Pract. (2013) 9:108–17. doi: 10.33698/NRF0159

[22] Gervaz P Bucher P Scheiwiller A Mugnier-Konrad B Morel P. The duration of postoperative ileus after elective colectomy is correlated to surgical specialization. Int J Color Dis. (2006) 21:542–6. doi: 10.1007/s00384-005-0050-0, PMID: 16267669

[23] Uebanso T Shimohata T Mawatari K Takahashi A. Functional roles of B-vitamins in the gut and gut microbiome. Mol Nutr Food Res. (2020) 64:e2000426. doi: 10.1002/mnfr.202000426, PMID: 32761878

[24] Guetterman HM Huey SL Knight R Fox AM Mehta S Finkelstein JL. Vitamin B-12 and the gastrointestinal microbiome: a systematic review. Adv Nut (Bethesda, Md). (2022) 13:530–58. doi: 10.1093/advances/nmab123, PMID: 34612492 PMC8970816

[25] Wang H Shou Y Zhu X Xu Y Shi L Xiang S. Stability of vitamin B12 with the protection of whey proteins and their effects on the gut microbiome. Food Chem. (2019) 276:298–306. doi: 10.1016/j.foodchem.2018.10.033, PMID: 30409598

[26] Wienhausen G Moraru C Bruns S Tran DQ Sultana S Wilkes H. Ligand cross-feeding resolves bacterial vitamin B(12) auxotrophies. Nature. (2024) 629:886–92. doi: 10.1038/s41586-024-07396-y, PMID: 38720071

[27] Lawrence RA Lawrence RM. Breastfeeding: a guide for the medical professional. Philadelphia: Elsevier Health Sciences (2021).

[28] Polegato BF Pereira AG Azevedo PS Costa NA Zornoff LAM Paiva SAR. Role of thiamin in health and disease. Nutr Clin Pract. (2019) 34:558–64. doi: 10.1002/ncp.10234, PMID: 30644592

[29] Singh M. Effect of thiamin deficiency on pancreatic acinar cell function. Am J Clin Nutr. (1982) 36:500–4. doi: 10.1093/ajcn/36.3.500, PMID: 6180623

[30] Donnino M. Gastrointestinal beriberi: a previously unrecognized syndrome. Ann Intern Med. (2004) 141:898–9. doi: 10.7326/0003-4819-141-11-200412070-00035, PMID: 15583247

[31] Smith TJ Johnson CR Koshy R Hess SY Qureshi UA Mynak ML. Thiamine deficiency disorders: a clinical perspective. Ann N Y Acad Sci. (2021) 1498:9–28. doi: 10.1111/nyas.14536, PMID: 33305487 PMC8451766

[32] Tallaksen CM Sande A Bøhmer T Bell H Karlsen J. Kinetics of thiamin and thiamin phosphate esters in human blood, plasma and urine after 50 mg intravenously or orally. Eur J Clin Pharmacol. (1993) 44:73–8. doi: 10.1007/BF00315284, PMID: 8436160

[33] Adams JF. Biological half-life of vitamin B12 in plasma. Nature. (1963) 198:200. doi: 10.1038/198200a0, PMID: 14010994

