# Supplementary material for: The combined use of vitamin B1 and vitamin B12 accelerates the recovery of gastrointestinal function after rectal cancer surgery
Source: Front Nutr. 2025 Oct 23;12:1658150. doi: 10.3389/fnut.2025.1658150 (PMC12588864; doi:10.3389/fnut.2025.1658150)
Supplement: Supplementary file 1 [file Table_1.docx]

**Supplementary Table 1.** Comparison of Clinicopathological characteristics Between Two Groups

| **Variable** | **B1B12 (n=123)** | **No (n=24)** | **P value** |
| --- | --- | --- | --- |
| **Sex** |  |  | 0.210 |
| Female | 40 (32.52) | 11 (45.83) |  |
| Male | 83 (67.48) | 13 (54.17) |  |
| **Age (year)** | 64.19 (11.26) | 60.96 (12.3) | 0.208 |
| **BMI (kg/m²)** | 22.71 (3.32) | 22.99 (3.1) | 0.707 |
| **ASA** |  |  | **<0.001** |
| I | 21 (17.07) | 0 (0) |  |
| II | 69 (56.10) | 5 (20.83) |  |
| III | 33 (26.83) | 19 (79.17) |  |
| **Smoke** |  |  | 0.730 |
| No | 88 (71.54) | 18 (75.00) |  |
| Yes | 35 (28.46) | 6 (25.00) |  |
| **Alcohol** |  |  | 1.000 |
| No | 104 (84.55) | 21 (87.50) |  |
| Yes | 19 (15.45) | 3 (12.50) |  |
| **Hypertension** |  |  | **0.049** |
| No | 71 (57.72) | 19 (79.17) |  |
| Yes | 52 (42.28) | 5 (20.83) |  |
| **CHD** |  |  | 0.308 |
| No | 106 (86.18) | 23 (95.83) |  |
| Yes | 17 (13.82) | 1 (4.17) |  |
| **Diabetes** |  |  | 1.000 |
| No | 99 (80.49) | 20 (83.33) |  |
| Yes | 24 (19.51) | 4 (16.67) |  |
| **Tumor distance (cm)** | 9 (7,12) | 8 (6,10.25) | 0.200 |
| **Preoperative hemoglobin (g/L)** | 119.76 (19.25) | 120.29 (16.47) | 0.899 |
| **Preoperative albumin (g/L)** | 38.74 (4.12) | 37.83 (4.11) | 0.324 |
| **Ileus** |  |  | 0.599 |
| No | 116 (94.31) | 24 (100) |  |
| Yes | 7 (5.69) | 0 (0) |  |
| **Neoadjuvant therapy** |  |  | 0.812 |
| No | 95 (77.24) | 18 (75.00) |  |
| Yes | 28 (22.76) | 6 (25.00) |  |
| **Neoadjuvant radiotherapy** |  |  | 0.531 |
| No | 103 (83.74) | 22 (91.67) |  |
| Yes | 20 (16.26) | 2 (8.33) |  |
| **Operation type** |  |  | 0.468 |
| Dixon | 110 (89.43) | 23 (95.83) |  |
| Miles | 13 (10.57) | 1 (4.17) |  |
| **Combined surgery** |  |  | 0.254 |
| No | 119 (96.75) | 22 (91.67) |  |
| Yes | 4 (3.25) | 2 (8.33) |  |
| **Time (min)** | 295 (249.5,349.5) | 287 (224.5,334) | 0.316 |
| **Blood loss** | 30 (20,50) | 20 (10,50) | **0.028** |
| **Enterostomy** |  |  | 0.290 |
| No | 90 (73.17) | 15 (62.50) |  |
| Yes | 33 (26.83) | 9 (37.50) |  |
| **Enterostomy type** |  |  | 0.151 |
| No | 90 (73.17) | 15 (62.50) |  |
| Ileum | 20 (16.26) | 8 (33.33) |  |
| Colon | 13 (10.57) | 1 (4.17) |  |
| **TNM stage** |  |  | 0.588 |
| I | 23 (18.70) | 7 (29.17) |  |
| II | 40 (32.52) | 6 (25.00) |  |
| III | 42 (34.15) | 9 (37.50) |  |
| IV | 18 (14.63) | 2 (8.33) |  |

Note. All values are presented as number (percentage) for categorical variables and as median (interquartile range, IQR) or mean (standard deviation, SD) for continuous variables, as appropriate. BMI, Body Mass Index. ASA, American Society of Anesthesiologists. CHD, Coronary Heart Disease. Tumor distance, Distance between tumor and anal verge. Miles is an abdominoperineal radical resection of rectal cancer, and Dixon is a transabdominal resection of rectal cancer. Bold p-values indicate statistical significance (*p* < 0.05).
